# A Case of Epicardial Epidermoid Cyst in a Crested Porcupine

**DOI:** 10.3390/ani14182706

**Published:** 2024-09-18

**Authors:** Alessia Mariacher, Valentina Galietta, Gianni Massai, Francesco Bruni, Giovanni Ragionieri, Claudia Eleni, Gianluca Fichi

**Affiliations:** 1 Istituto Zooprofilattico Sperimentale delle Regioni Lazio e Toscana, UOT Toscana Sud, Viale Europa 30, 58100 Grosseto, Italy; 2 Istituto Zooprofilattico Sperimentale delle Regioni Lazio e Toscana, UOC Diagnostica Generale, Via Appia Nuova 1411, 00178 Rome, Italy; valentina.galietta@izslt.it (V.G.); claudia.eleni@izslt.it (C.E.); 3 Istituto Zooprofilattico Sperimentale delle Regioni Lazio e Toscana, UOT Toscana Sud, Viale Toselli 12, 53100 Siena, Italygianluca.fichi@izslt.it (G.F.); 4 Veterinary Practitioner, 53100 Siena, Italy

**Keywords:** choristoma, heart, heterotopia, histopathology, *Hystrix cristata*

## Abstract

**Simple Summary:**

One male adult crested porcupine was found moribund in the province of Siena (Tuscany, Central Italy), and died soon after being recovered by a wildlife rescue service. At necropsy, a rounded nodule was noted on the surface of the heart. Differential diagnoses included abscess, systemic tuberculosis, parasitic cyst, and neoplasia. Histology was performed on the lesion, revealing a cystic formation in the epicardium. The cyst was lined by stratified squamous epithelium and was filled with lamellar keratin without hair shafts. The lesion was diagnosed as an epicardial epidermoid cyst (EC). EC are most commonly found in the skin, both in human and animal patients, although rarely they can occur in various internal organs. However, cardiac EC has not been reported before in animals. To the best of our knowledge, this is the first report of EC in a wild animal species.

**Abstract:**

The crested porcupine (*Hystrix cristata*) is present in central Italy with an estimated population of 1800 individuals. Despite the local abundance, little data are available on the diseases affecting free-ranging individuals. We describe a case of an epidermoid cyst (EC) in a male adult porcupine found in the municipality of Sovicille, province of Siena (Tuscany). At necropsy, a firm rounded nodule was noted on the left ventricle wall. Histological examination revealed a cystic formation lined by stratified squamous epithelium. The cyst was filled with lamellar keratin, while hair shafts were not present. The adjacent epicardium was infiltrated by lymphoplasmacytic cells in reaction to the rupture of the cyst with the spilling of keratinaceous debris. The lesion was diagnosed as a ruptured epicardial epidermoid cyst. EC are most commonly found in the skin, both in human and animal patients, though infrequently, they can occur in any internal organ. Cardiac EC has not been reported in domestic animals, and this is the first report of EC in a wild animal species. Clinical veterinarians should consider the possibility of similar cardiac lesions in captive subjects since the long lifespan of these rodents could allow the growth of the cyst with the compression of the adjacent tissues.

## 1. Introduction

The crested porcupine (*Hystrix cristata*) outside of Africa is currently present in mainland Italy, Sicily, and Sardinia [1,2]. In the Italian peninsula, the species distribution is continuous in the central regions, while the distribution in the northern and southern regions is more fragmentary [3]. Species abundance in Tuscany was estimated to be 1803 individuals by Franchini and colleagues [4] through presence data obtained from camera traps, direct observations, and road-killing casualties.

Despite the local abundance and widespread presence in the Italian territory, little data are available on the health status of the crested porcupine population or on the common medical issues that can affect free-ranging individuals. It is reported that adult or subadult individuals are more frequently hospitalized in wildlife rescue centers, while a lower number of porcupettes gets rescued [5]; nonetheless, there are no reports of individual clinical assessments or pathologic data of the animals rescued in these facilities. The majority of the published works so far refer to parasitological studies in injured or road-killed porcupines, without the mention of clinical- or pathological- associated pictures. Cavallero et al. [6] performed a morphometrical and molecular analysis of *Trichocephalus* spp., in an attempt to identify distinct lineages infecting the species. Coppola and colleagues [7] reported a high prevalence of *Giardia* spp. on fecal samples, along with other less represented parasites (*Cryptosporidium* spp., *Trichocephalus* spp., gastrointestinal strongyles, Capillariids, and coccidian oocysts). In a study based on external examination only, 24% of the observed porcupines were infested by different species of fleas and/or ticks, with a higher prevalence for *Ixodes ricinus* and *Pulex irritans* [8], while a single case of sarcoptic mange was described with both gross and microscopic description in a female porcupine from southern Italy [9]. In addition, a case of a traumatic wound infested by *Calliphora vicina* larvae was reported in a juvenile female [10].

A case of systemic tuberculosis by *Mycobacterium bovis* was described in an adult male porcupine: at necropsy, pleuropneumonia with nodular lesions and mediastinal lymphadenitis were noted. Histologically, the lesions consisted of diffusive granulomatous foci, surrounded by epithelioid cells and giant multinucleated Langhans-type cells containing rod-shaped Z-N positive bacteria. The porcupine was regarded as a spill-over host in this instance [11]. A histopathological study was also conducted in a case of disseminated granulomatous pneumonia in a juvenile female porcupine, where cystic structures were observed, consistent with adiaspiromycosis by *Emmonsia crescens*, as further confirmed by a PCR assay [12]. *L. interrogans* serogroup Pomona serovar Pomona was isolated from one case of interstitial nephritis in a porcupette, leading the authors to hypothesize that the porcupine may represent a new host for this zoonotic bacterium [13].

As for viral infections, an outbreak of Encephalomyocarditis Virus (EMCV) infection in a captive setting and an RT-PCR positivity for Hepatitis E Virus in wild porcupines were also reported [14,15]. Gross and microscopic lesions were only described for EMCV, and consisted of interstitial myocarditis with grossly visible necrotic foci, and lymphocytic meningoencephalitis [14].

In this work, we describe the gross and microscopic findings that were found during the analysis of a specimen of an adult male porcupine, which was found dead in the province of Siena (Tuscany, Central Italy) in the spring of 2024. The examination of the subject revealed a small cardiac mass, whose histological diagnosis was a ruptured epicardial epidermoid cyst (EC). A description of the lesion and similar findings described in the literature in different animal species are provided.

## 2. Materials and Methods

In April 2024, a free-ranging crested porcupine was found moribund on the roadside in the municipality of Sovicille, in the province of Siena (Tuscany, Central Italy) (geographic coordinates: 43°29′05.5″ N, 11°00’05.3″ E). The animal was recovered by a wildlife rescue veterinary service but died in two days. The carcass was refrigerated at 4 °C and delivered within 12 h to the Istituto Zooprofilattico Sperimentale of Latium and Tuscany, Siena, for the investigation of the cause of death.

The autopsy was performed by veterinarian pathologists within 4 h, following the international standard procedures [16]. The carcass was identified following the literature description [17], and examined for any external abnormality and post-mortem lesions. After dissection, the internal organs were examined for any gross lesion. Parasitological, mycological, and bacteriological examinations were performed from external lesions, while internal organs, such as the lungs, spleen, liver, kidney, and pericardium, were submitted for bacteriological examination. Feces were collected and conserved at 4 °C for parasitological examination.

Inocula from skin lesions and subcutaneous lesions, the lungs, spleen, liver, kidney, and pericardium were taken with a sterilized loop and plated onto Blood Agar and MacConkey Agar plates (IZSLT, Rome, Italy), and aerobically and anaerobically incubated at 37 °C for 24 h. Taxonomical analyses according to Bergey’s Manual of Determinative Bacteriology [18] were conducted on the developed bacterial colonies. Briefly, the morphological characteristics of the colonies, Gram staining, and catalase and oxidase results were considered. For bacterial identification, the biochemical reactions were performed on API test strips (Biomerieux TM, Marcy-l’Étoile, France). Inocula from the skin were taken with a sterilized loop and plated on Sabouraud Dextrose Agar and incubated at 30 °C for 7 days, for mycological examination. After 7 days, the morphology of the fungi colonies was examined, and cell observation was performed through microscopic examination after Cotton Blue Lactophenol fresh staining [19]. Parasitological external and internal investigations were conducted, including the gross examination, and the examination of skin scraping samples, using a dissecting microscope after digestion through 10% potassium hydroxide [20].

A fecal sample was collected from the rectum and qualitatively examined according to previously published methods [21]. Samples of skin, subcutaneous lymph nodes, and heart were collected during the necropsy, fixed in 10% neutral buffered formalin, embedded in paraffin wax, sectioned at 4 μm, stained with Haematoxylin and Eosin (HE) and examined histologically under a light microscope.

## 3. Results

**Figure 1:**
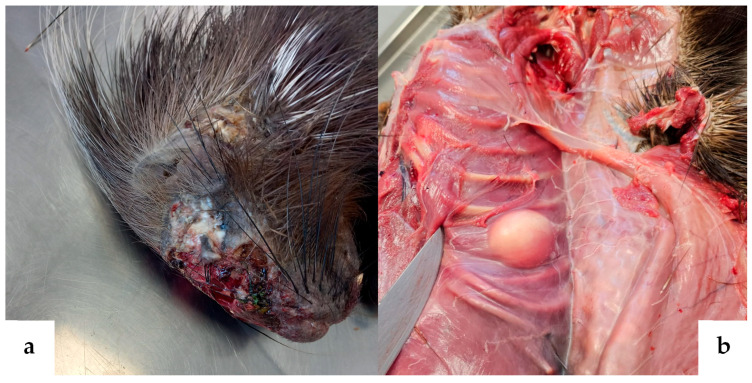
(**a**) Crested porcupine, head. Full-thickness ulceration of the skin in the frontal, maxillary, and nasal regions; (**b**) crested porcupine, right rib cage. After the removal of the skin (reflected on the right side of the image), a rounded mass of about 4 cm in diameter with a pasty consistency is observed on the caudal right rib cage, which at the cut surface is diagnosed as a non-calcified abscess.

**Figure 2:**
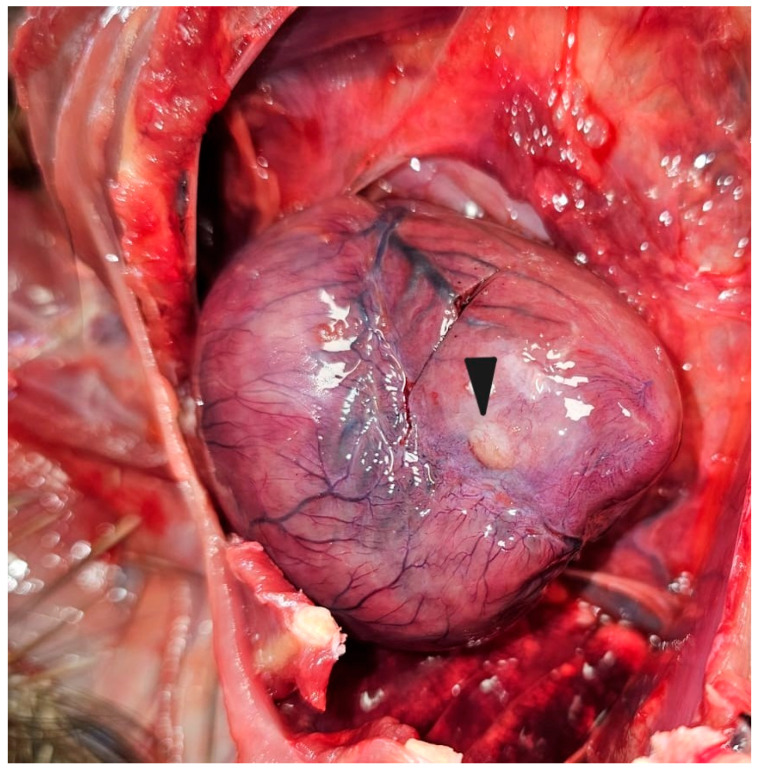
Crested porcupine, heart. A rounded whitish mass of approximately 1 cm in diameter is visible on the left ventricle wall (arrowhead).

The subject under examination was identified as an adult male porcupine. The carcass was well preserved and free from colonization by cadaveric entomofauna. At necropsy, an external inspection revealed the presence of multiple wide skin erosions and ulcers, particularly severe in the maxillary and frontal regions (Figure 1a). Several abscesses in the subcutis and skeletal muscles were noted. Some abscesses formed fistulous tracts emerging at the neck and groin regions. After skin removal, another abscess was observed on the external surface of the right chest wall (Figure 1b). Generalized lymphadenopathy with enlarged and hyperaemic lymph nodes was present. In the thoracic cavity, chronic bilateral pneumonia with the consolidation of lung lobes was observed, along with pericarditis and myocarditis. In addition, on the epicardium of the left ventricle, a firm, rounded, raised whitish nodule, approximately 1 cm in size, was noted (Figure 2).

Concerning bacteriology tests, *Staphylococcus aureus*, and *Streptococcus agalactiae* were identified after culturing from cutaneous lesions, while *S. agalactiae* was also isolated from abscesses in the subcutis and skeletal muscles, and in the intracardial blood clot. No pathogenic dermatophytes or ectoparasites were evidenced from skin lesions. At the parasitological examination of the carcass and feces, no internal parasites or eggs/oocysts were found. Differential diagnoses for the heart nodule, at this time, included abscess, nodular mycobacterial lesion, parasitic cyst, and neoplasia.

**Figure 3:**
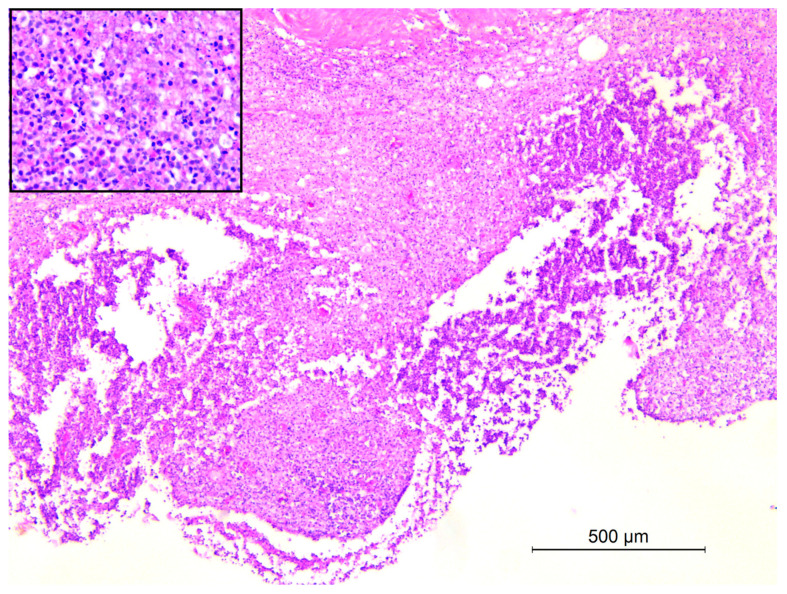
Subcutis adjacent to a skin abscess, crested porcupine. Severe diffuse pyogranulomatous panniculitis. Inset: detail of lesion with cellular debris, numerous degenerate neutrophils, epithelioid macrophages, and fewer lymphocytes and plasma cells (H&E, 5×).

Histologically, the skin and subcutaneous tissue exhibited severe diffuse deep pyogranulomatous dermatitis and panniculitis (Figure 3), with numerous colonies of cocci. A similar inflammatory pattern was observed in the lymph nodes, presenting as severe multifocal pyogranulomatous lymphadenitis.

**Figure 4:**
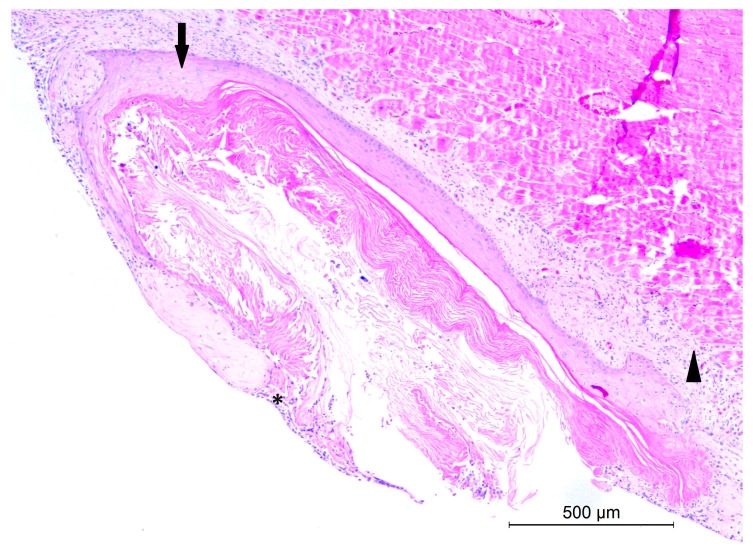
Epidermoid cyst, heart, crested porcupine. The histological section shows a cystic structure growing in the epicardium (arrowhead), with a wall of well-differentiated stratified squamous epithelium (arrow), which appears discontinuous with keratin spilling (asterisk) and moderate lymphocytic and neutrophilic infiltration (H&E, 5×).

The histological examination of the heart revealed a mild diffuse lymphoneutrogranulocytic epicarditis and multifocal myocarditis. In the epicardium, a cystic formation was also observed. The cyst was lined by a well-differentiated stratified squamous epithelium (Figure 4) and contained lamellar keratin. Hair shafts were not present in the lumen of the cyst. The wall appeared discontinuous, with keratin spilling outward from the cyst. Additionally, a moderately loose fibrovascular stroma surrounding the cyst was diffusely infiltrated by lymphocytes, plasma cells, and a few neutrophils. The lesion was diagnosed as a ruptured epidermoid cyst.

## 4. Discussion

The porcupine specimen examined in this case report was primarily affected by diffuse skin lesions (pyogranulomatous dermatitis, ulcers, and abscesses) and generalized lymphadenitis. *S. aureus* and *S. agalactiae* were cultured from cutaneous lesions, a finding that is difficult to interpret since these bacteria are commensal skin bacteria and opportunistic pathogens in terrestrial mammals [22]. However, since *S. agalactiae* was also isolated from the intracardial blood clot, it is possible that the subject was suffering from septicemia related to severe skin lesions. Multiple skin abscesses were previously described in a case of sarcoptic mange in a porcupine, but in this case, we excluded this diagnosis based on external findings, a negative search for ectoparasites, and the results of the histological examination, which did not reveal hyperplasia, hyperkeratosis, or mites [9]. The cause of the observed lesions cannot be determined unequivocally; however, we hypothesize that the subject may have been affected by episodes of intraspecific aggression with consequent traumatic and penetrating lesions from quills, as could be suggested, for example, by the presence of an abscess in the rib cage [23]. However, the most interesting aspect of the case is the finding of a peculiar lesion at the cardiac level.

The presence of differentiated noncardiac tissue in the heart is commonly referred to as cardiac heterotopia [24]. Intracardiac heterotopias are uncommon and, in almost all the cases reported in human medicine, the main aberrant tissue is of epithelial origin [25]. One type of cardiac heterotopia commonly reported in veterinary medicine is bovine myocardial epithelial inclusions, which consist of single to multiple foci of squamous to cuboidal epithelial cells, usually located in the left ventricle wall [26,27]. When heterotopic tissue is organized in discrete masses, it is rather referred to as a choristoma.

Epidermoid cysts (ECs) are rare, congenital, slow-growing, benign masses that arise from heterotopic epithelium. Grossly, these masses are usually described as rounded to oval, firm or with a soft core, with a smooth surface, and well circumscribed [28]. The macroscopic appearance can lead to a differential diagnosis of abscess [29], parasitic cyst, or malignancy [30]. At the histopathologic examination, EC is lined by a well-differentiated stratified squamous epithelium that resembles a normal epidermis and can contain the intraluminal breakdown products of desquamated epithelial cells, such as keratin, cholesterol crystals, or granules [31,32,33].

ECs are most commonly found in the skin, both in human and animal patients, though infrequently they can occur in any internal organ. Non-integumentary EC in human patients has been described in the brain, spleen, and gonads, with the cardiac location being a rarer occurrence [34,35]. In veterinary medicine, the most commonly reported locations are intracranial, intraspinal, and intraosseous, although many other organs, both in the thoracic and abdominal cavity, have been described as the possible sites of EC development, such as the rumen, intestine, spleen, and gonads (Table 1). However, cardiac EC has not been reported before in domestic animals, and there are no reports of EC in wild animal species.

Various hypotheses have been advanced regarding the origin of EC, which could be congenital (from the inclusion of ectodermal elements during embryological development) or acquired later in life, possibly as a result of penetrating injuries (including surgical procedures) or foreign body reactions. These latter events would allow the implantation of epidermal fragments in locations favorable to their growth [41,51]. In our case, it is not possible to determine a congenital or acquired origin of the described lesion, since we have no elements of the animal’s history, nor from comparison with other cases, to support one hypothesis or the other. However, considering the observed skin lesions and the presence of multiple subcutaneous abscesses, we cannot exclude that porcupine quills, which can reach up to 30–40 cm in length [71], could have acted as foreign bodies in previous intraspecies conflicts.

It is possible that the mass had been growing over a certain time due to desquamation leading to an accumulation of keratinaceous material at the center of the cyst [34]. Furthermore, in the present case, the cyst wall appeared discontinuous, with keratin spilling outward and causing a lymphoplasmacytic and neutrogranulocytic infiltrate in the surrounding epicardium. Also in some previously reported cases, the EC wall was ruptured, resulting in the microscopic pictures of granulomatous nature consistent with foreign body reactions [53,59,67]. Even when the cyst wall is apparently intact, mild inflammation of the stroma surrounding the EC has been described [36,41,62], while the inflammatory component is usually absent in simple epithelial cysts [72].

The clinical significance of epicardial EC in the present case is doubtful. Since the mass did not occupy a significant space in the pericardial sac, and apparently it was not limiting the heart ventricular capacity, this may represent an incidental finding. However, since the porcupine has one of the longest lifespans among rodents (up to 28 years in captivity) [73], a space-occupying lesion in the heart could generate cardiovascular dysfunctions if allowed sufficient time for its growth. The possibility of similar cardiac lesions in captive subjects should be considered by wildlife or zoo veterinarians, since based on the lifespan of these rodents, the growth of the EC with the compression of the adjacent tissues could be expected.

## 5. Conclusions

Despite its widespread presence in Italy and its high protection status, the crested porcupine has been scarcely studied from a clinical and pathological point of view. In this report, we describe the case of a porcupine in which, in addition to the presence of a generalized infection by Gram-positive bacteria, a small whitish mass was observed in the epicardium of the left ventricle. The mass was histologically diagnosed as an epidermoid cyst. To the best of the authors’ knowledge, this is the first case of cardiac epidermoid cyst in an animal patient and the first description in a wild species. The possibility of the development and growth of epidermoid cysts in captive porcupines, or in rescued subjects with a history of previous intraspecific conflicts involving the penetration of quills, should be considered by clinical veterinarians, since the long lifespan of these rodents may allow for the growth of EC with the compression of the adjacent tissues.

## Figures and Tables

**Table 1:** Anatomic locations of non-cutaneous epidermoid cysts reported in different animal species.

Animal Species	Anatomic Location	Reference
Dog	Nasopharynx	[36,37]
	Brain	[31,38,39,40,41,42,43,44]
	Spinal cord	[32,45,46,47,48]
	Diploe	[49]
	Bone	[50,51,52,53,54]
	Pelvic retroperitoneal space	[55]
	Intestine	[56,57]
	Spleen	[30]
	Mammary gland	[58]
Cat	Brain	[59]
	Intestine	[60]
	Lung	[61]
Cattle	Ovary	[29]
	Rumen	[62]
Rats, Mice	Brain and spinal cord	[63,64]
Horse	Intracranial	[65,66]
	Intra-articular	[67]
	Bone	[68,69]
Rabbit	Testis	[70]

## Data Availability

The original contributions presented in the study are included in the article; further inquiries can be directed to the corresponding author.
